# The Prevention of Disease

**Published:** 1902-10

**Authors:** Ed Braden

**Affiliations:** San Antonio, Texas


### The Prevention of Disease.

All scientific authorities agree that the human waste products and filth are the most prolific source of disease-breeding bacteria.

The sewer is the modern means of disposal. No sane man defends the cesspool or open ditch as a safe means. The word "sanitary" when applied to sewer is misleading.

There is no such thing as sanitary sewer. You may have a sanitary home if the plumbing is perfect, but no amount of flushing, scouring or cleaning can make a public sewer sanitary. A system of sewers not utilized by all the people is a crime against the taxpayers—a waste of public funds. The object of scientific plumbing is to quarantine your home against sewer air by a system of house drainage and the proper ventilation of traps and pipes. The true mission of the plumber, then, is to keep dangerous sewer air out of the home. The ground work of house plumbing and drainage being concealed from view offers an almost limitless field in which dishonesty and incompetency can operate unchallenged. The grave symptoms of this condition cause you no alarm. You pay your doctor, you bury your dead and you pay plumber's repairs with no very good grace, ignorant of the fact that it is within your power to change or greatly mitigate all this.

Demand scientific plumbing. Select fixtures with care. The cheapest is not the best. Take proper care of your fixtures. Remember the plumber's business is to prevent disease; the physician's to cure.

Very respectfully,

ED BRADEN, JR.,
San Antonio, Texas.

